# Immunogenicity Assessment of Lipegfilgrastim in Patients with Breast Cancer Receiving Chemotherapy

**DOI:** 10.1155/2016/9248061

**Published:** 2016-06-23

**Authors:** Linglong Zou, Anton Buchner, Martin Roberge, Patrick M. Liu

**Affiliations:** ^1^Global Bioassays and Technology, Teva Pharmaceuticals, Inc., 145 Brandywine Parkway, West Chester, PA 19380, USA; ^2^Merckle GmbH, Graf-Arco-Straße 3, 89079 Ulm, Germany; ^3^CIRION BioPharma Research Inc., 3150 rue Delaunay, Laval, QC, Canada H7L 5E1

## Abstract

Lipegfilgrastim is a long-acting, once-per-cycle, glycopegylated recombinant granulocyte colony-stimulating factor (G-CSF) used to prevent neutropenia in patients receiving myelosuppressive chemotherapy. This integrated analysis examined the immunogenicity of lipegfilgrastim and its potential clinical impact in two double-blind randomized studies (phases II and III) of patients with breast cancer receiving chemotherapy. Serum samples were analyzed using sequential assays for screening, confirmation, antibody titer, and characterization of antidrug antibodies (ADA). Neutropenia-related efficacy measures were reviewed for each ADA-positive patient. Among 255 patients receiving lipegfilgrastim (154 in phase II, 101 in phase III) and 155 patients receiving pegfilgrastim (54 in phase II, 101 in phase III), the incidence of treatment-emergent ADA was low and similar between the lipegfilgrastim (phase II: 1.3%; phase III: 1.0%) and pegfilgrastim (phase II: 1.9%; phase III: 1.0%) arms. None of the treatment-emergent ADA-positive samples exhibited neutralizing activity against lipegfilgrastim, pegfilgrastim, or glycosylated G-CSF in a cell-based neutralizing antibody assay. No changes were observed in neutropenia-related efficacy measures among ADA-positive patients, and no treatment-related hypersensitivity or anaphylaxis occurred. These results indicate that there is no apparent impact of ADA on lipegfilgrastim efficacy and safety.

## 1. Introduction

Granulocyte colony-stimulating factor (G-CSF) is an endogenous growth factor that promotes neutrophil production, maturation, survival, and activity [1]. Recombinant G-CSFs, such as filgrastim and pegfilgrastim, are used commonly for the prevention and treatment of neutropenia in patients receiving myelosuppressive chemotherapy [2–4].

Filgrastim requires daily administration to maintain therapeutic levels because of its relatively short half-life. Conjugating filgrastim to polyethylene glycol (PEG; pegylation yielding pegfilgrastim) reduces renal clearance and extends the drug's half-life such that it need be administered only once per chemotherapy treatment cycle, with efficacy and safety comparable to those of daily filgrastim [5–7].

Lipegfilgrastim (Lonquex; Teva Pharmaceuticals Ltd.) is a recombinant human G-CSF that is glycopegylated in a site-specific manner, resulting in greater structural homogeneity, with pharmacological properties slightly different from those of pegfilgrastim in healthy volunteers. Specifically, lipegfilgrastim provided a longer-lasting increase in absolute neutrophil count (ANC) compared with pegfilgrastim at an equivalent dose, without increasing the peak ANC values [8]. The noninferiority of lipegfilgrastim to pegfilgrastim in the treatment of severe neutropenia was demonstrated in a randomized, double-blind, active-controlled, phase III trial evaluating the efficacy and safety of lipegfilgrastim in 202 chemotherapy naive patients with breast cancer [9]. Lipegfilgrastim was approved in the European Union in 2013 as once-per-cycle, fixed-dose prophylaxis for severe neutropenia.

Immunogenicity is a potential concern for any biological product, and its assessment is one of the most critical elements for the development of such products. Antidrug antibody (ADA) production, as an unwanted immune response due to product immunogenicity, may lead to serious safety consequences that manifest as hypersensitivity responses such as anaphylaxis and development of cross-reactive neutralizing antibodies (NAbs) to endogenous proteins [10, 11]. Recombinant G-CSFs, including filgrastim and pegfilgrastim, have been shown to elicit ADA in a minority of patients [12, 13].

The objective of this analysis was to assess the immunogenicity of lipegfilgrastim and its potential clinical impact using data from phase II dose-finding trial and phase III noninferiority trial conducted with patients with breast cancer receiving chemotherapy.

## 2. Methods

### 2.1. Study Design and Treatments

Immunogenicity assessments were performed on blood samples collected during two independent clinical studies [9, 14]. The first study was a phase II, double-blind, randomized, dose-optimization study that evaluated the efficacy, safety, pharmacokinetics, and immunogenicity of drug treatments in 208 breast cancer patients undergoing myelosuppressive chemotherapy. Patients were assigned 1 : 1 : 1 : 1 to receive lipegfilgrastim (3.0, 4.5, or 6.0 mg administered via subcutaneous [SC] injection) or pegfilgrastim (6.0 mg SC) once per cycle while undergoing chemotherapy with intravenous doxorubicin 60 mg/m^2^ and docetaxel 75 mg/m^2^ [14]. The second study was a phase III, double-blind, randomized, noninferiority study in which 202 patients with breast cancer received either lipegfilgrastim (6.0 mg SC) or pegfilgrastim (6.0 mg SC) once per cycle while undergoing the same chemotherapy regimen [9].

In both studies, patients received intravenous doxorubicin/docetaxel administered on day 1 of four 21-day cycles. Lipegfilgrastim or pegfilgrastim was administered on day 2 of each cycle (i.e., 24 hours after chemotherapy was administered).

Blood samples were collected at several time points in each study: at baseline, prior to each chemotherapy cycle, at the end of treatment (day 85), and on posttreatment follow-up days 180 and 360.

### 2.2. Study Populations

Eligible patients (≥18 years of age) had a diagnosis of stage II, III, or IV breast cancer, were chemotherapy naive, had a baseline ANC of at least 1.5 × 10^9^/L and a platelet count of 100 × 10^9^/L or greater, and had an Eastern Cooperative Oncology Group performance status of 2 or less [9, 14]. Exclusion criteria included known hypersensitivity to filgrastim or pegfilgrastim or exposure to those agents prior to randomization, prior malignancy within 5 years, radiation therapy within 4 weeks of randomization, or long-term use of oral corticosteroids.

The overall population comprised 208 patients from the phase II study (54 pegfilgrastim, 154 lipegfilgrastim) and 202 patients from the phase III study (101 in each treatment arm), for a total of 410 patients. Patient demographics and baseline clinical characteristics were matched between treatment groups within each study and have been reported elsewhere [9, 14]. The mean ages were similar between the lipegfilgrastim (51.4 years) and pegfilgrastim (50.5 years) groups. All patients were white, and all but three were female.

### 2.3. Immunogenicity Assays

**Figure 1:**
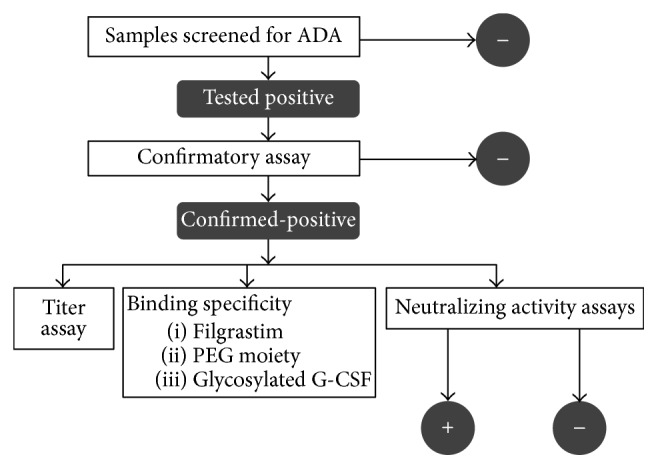
Sequential approach to assessing immunogenicity.

A sequential cascade of validated assays was used to analyze ADA against lipegfilgrastim in patient serum samples (Figure 1). All samples were first tested in the screening assay. Samples screening positive were then analyzed using a confirmatory assay. After ADA confirmation, samples were characterized to determine binding specificity, antibody titers, and neutralizing activity.

#### 2.3.1. Screening Assay

**Figure 2:**
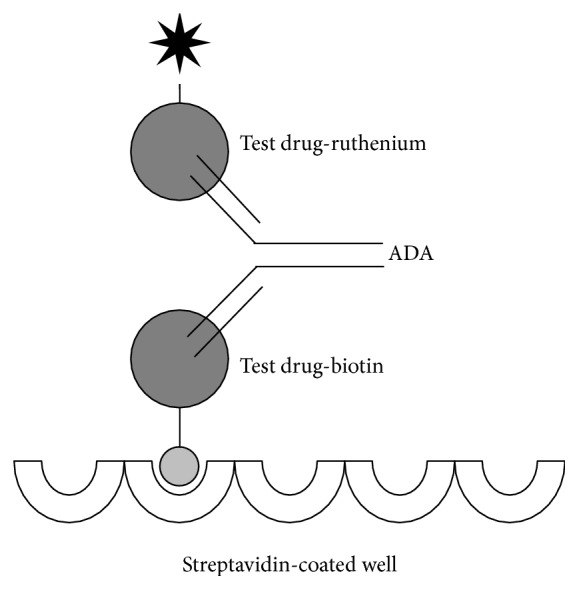
Schematic presentation of the electrochemiluminescent bridging immunoassay. Patient samples were diluted at the minimum required dilution, mixed with biotin- and ruthenium-conjugated test drug (lipegfilgrastim or pegfilgrastim) and the complex formed by antidrug antibodies (ADA). The drug conjugates were captured on a streptavidin-coated assay plate. In the presence of a read buffer containing tripropylamine and upon application of an electrical potential, the ruthenium tag emits light.

The screening assay used a ligand-binding principle in an electrochemiluminescent bridging format (Figure 2). Briefly, the bivalent property of ADA allows for simultaneous binding of a capture reagent (biotin-labeled lipegfilgrastim or pegfilgrastim) and a detection reagent (ruthenium-labeled lipegfilgrastim or pegfilgrastim). In the presence of a read buffer containing tripropylamine, an electrical current causes the captured ruthenium to emit measureable light. Signal was measured using a Sector Imager 6000 Analyzer (Meso Scale Discovery). The relative sensitivity of the screening assay was 10 ng/mL ADA to lipegfilgrastim and 45 ng/mL ADA to pegfilgrastim, determined using affinity-purified rabbit anti-lipegfilgrastim and rabbit anti-pegfilgrastim antibodies, respectively, as surrogate positive controls. Assay signals from a panel of samples from study drug-naive breast cancer patients were used for the determination of the assay cut-point factor, which in conjunction with the negative control was used to calculate the cut-point for each assay run. Cut-point factors with a false-positive rate set at 5% were determined using statistical methodology described elsewhere [15, 16]. A sample with an assay signal at or above the cut-point was considered as having screened positive.

#### 2.3.2. Confirmatory Assay

Samples that tested positive in the screening assay were subsequently analyzed in an immunocompetition assay for confirmation. Binding was measured with and without the study drug (lipegfilgrastim or pegfilgrastim) in solution phase, and the ratio of binding signal was calculated and expressed as a percentage of signal inhibition. A cut-point with a false-positive rate set at 1% was determined for each drug using commercially available serum samples from treatment-naive cancer patients in accordance with statistical methods [15, 16]. Samples were identified as confirmed-positive if the percentage of signal inhibition was greater than or equal to the confirmatory cut-point for lipegfilgrastim (28.5%) or pegfilgrastim (28.2%).

#### 2.3.3. Characterization Assays

Confirmed-positive samples were subsequently characterized to evaluate the ADA binding specificity. Binding signals were determined in the presence of unlabeled filgrastim, the PEG portion of lipegfilgrastim (cPEG), or glycosylated G-CSF (glycoG-CSF; Granocyte®; Chugai Pharma), which is an analog of endogenous G-CSF. The cut-points (% signal inhibition) for binding specificity were determined statistically (with false-positive rate set at 1%), using commercially available serum samples from treatment-naive cancer patients in accordance with statistical methodology [15, 16], to be 22.0% for filgrastim, 19.4% for cPEG, and 17.5% for glycoG-CSF.

All confirmed-positive serum samples were also subjected to a semiquantitative titer assay. The titer was defined as the logarithm-transformed highest dilution factor resulting in a signal at the screening cut-point.

#### 2.3.4. NAb Assays and Clinical Assessment

Neutralizing activity was assessed in the confirmed-positive ADA samples using a cell-based proliferation assay that tested the ability of the serum samples to inhibit various G-CSF (glycoG-CSF, lipegfilgrastim, or pegfilgrastim) stimulated proliferation of NSF-60 cells in vitro measured using WST-1 reagent. Samples were also tested in the absence of any G-CSF inducer to detect the presence of nonspecific cell growth that could result in false-negative neutralizing activity. Samples that inhibited any G-CSF inducers in the NAb assay underwent specificity testing in which proliferation was stimulated by murine interleukin-3, an inducer not specific to G-CSF activity; neutralization of this proliferation indicated nonspecific inhibition.

The cut-point for positivity was defined as an optical density (OD) ratio (OD sample : OD viability control) less than or equal to 0.824 for lipegfilgrastim inducer, 0.761 for pegfilgrastim inducer, and 0.821 for glycoG-CSF inducer. These cut-points were established statistically with the false-positive rate set at 5%. A cut-point with a multiplicative correction factor of 1.402 (1% false-positive rate) was established in accordance with statistical methods [15, 16] using commercially available serum samples from treatment-naive cancer patients when samples were tested in the absence of inducer.

Clinical measures were examined for all patients with confirmed-positive samples of ADA to lipegfilgrastim or pegfilgrastim for a possible correlation between the presence of ADA and the potential clinical impact of immunogenicity.

### 2.4. Efficacy Measurements

The primary efficacy measure was the duration of severe neutropenia (DSN), defined as the number of days with grade 4 neutropenia (ANC < 0.5 × 10^9^/L) in each treatment cycle. A secondary efficacy measure was the incidence of febrile neutropenia, defined as axillary body temperature greater than 38.5°C for more than 1 hour and ANC less than 0.5 × 10^9^/L across all cycles. Additional measures included ANC area under the concentration-time curve, maximum ANC, and mean depth of the ANC nadir.

## 3. Results

### 3.1. ADA Incidence

A total of 208 patients (54 pegfilgrastim and 154 lipegfilgrastim) were investigated in the phase II study and 202 patients in the phase III study. Results from the patients with confirmed ADA are summarized in Table 1. In the phase II study, 2 of the 154 lipegfilgrastim-treated patients (Table 1, patients 1 and 2) had treatment-emergent ADA, representing an incidence of 1.3%. These patients had confirmed-positive samples at a single postdose time point (day 85 or day 360), indicating that the response was transient. There were seven patients with predose ADA, including three with positive samples at both baseline and postdose time points (Table 1, patients 3–5) and four with positive samples at baseline only (Table 1, patients 6–9). One of 54 pegfilgrastim-treated patients (Table 1, patient 10) had treatment-emergent ADA, representing an incidence of 1.9%. In this patient, the ADA-positive sample occurred only at day 85. There were two patients in the pegfilgrastim group with positive ADA samples observed at baseline only (Table 1, patients 11 and 12).

In the phase III study, 1 of the 101 lipegfilgrastim-treated patients (Table 1, patient 13) had treatment-emergent ADA, with positive samples at days 180 and 360. This reflects an ADA incidence of 1.0%. There were three patients with predose ADA, including one who had a positive sample at baseline only (Table 1, patient 16) and two who had positive samples at both baseline and at least a postdose time point (Table 1, patients 14 and 15). Among 101 pegfilgrastim-treated patients in this study, one had treatment-emergent ADA, with a positive postdose sample at day 360 only (Table 1, patient 17). This also represents an ADA incidence of 1.0%. Six patients (Table 1, patients 18–23) had predose ADA, with positive samples at baseline only.

### 3.2. ADA Characterization and Titer

The binding specificity and titer of ADA were determined in samples from lipegfilgrastim-treated patients with predose or treatment-emergent ADA. Twenty-two confirmed-positive samples from nine lipegfilgrastim-treated patients in the phase II trial were tested using filgrastim, cPEG, or glycoG-CSF competitors to identify ADA binding specificity for the G-CSF moiety of lipegfilgrastim, the PEG moiety of lipegfilgrastim, and endogenous G-CSF, respectively (Table 2).

As noted above, only two patients in the lipegfilgrastim-treated group exhibited treatment-emergent ADA. One had a positive sample on day 85, with an antibody titer of 0.6 against cPEG only (Table 2). The other patient had the positive sample on day 360. The antibody titer was undetectable in this sample and showed no recognition of filgrastim, glycoG-CSF, or cPEG. Five of the seven remaining patients showed predose ADA; two of these five had positive postdose ADA samples as well. One of these two patients had antibodies recognizing filgrastim and glycoG-CSF without detectable titer throughout the duration of the study. The other patient had antibodies recognizing filgrastim and cPEG whose titer diminished over time from 0.9 to 0.3. The remaining three of these five patients had predose antibodies recognizing filgrastim and cPEG but no detectable postdose antibody titer.

From the phase III study, nine confirmed-positive samples from four patients underwent the same characterization and titer assays. One lipegfilgrastim-treated patient with a confirmed-positive sample had possible treatment-emergent antibody induction, recognizing filgrastim and glycoG-CSF, but not cPEG, on both day 180 and day 360 (Table 2). The sample also showed antibody titers of 1.2 and 2.1 for days 180 and 360, respectively. The remaining three patients had confirmed-positive samples at baseline (i.e., predose ADA). One of these patients had antibodies recognizing cPEG at baseline, with a titer of 0.6 that increased to 1.2 in cycle 2 but diminished to undetectable levels by day 85. Another patient had antibodies recognizing filgrastim and glycoG-CSF at baseline, with a titer of 2.1; the antibodies for glycoG-CSF persisted to cycle 2, with a titer of 1.8. The third patient had antibodies against filgrastim and cPEG, with a titer of 0.9 and no detectable postdose antibodies.

### 3.3. Neutralizing Activity of ADA

Among patients identified as having treatment-emergent ADA, no postdose sample from either treatment group in each study tested positive for NAb activity against lipegfilgrastim, pegfilgrastim, or glycoG-CSF in the cell-based neutralizing antibody assay.

### 3.4. Clinical Impact Assessments

Clinical efficacy measures for patients with confirmed-positive, treatment-emergent ADA are summarized in Table 3. Neither ANC nor DSN values changed in these patients after initiation of chemotherapy, and no patient experienced febrile neutropenia. No drug-related events with the Medical Dictionary for Regulatory Activities preferred term “drug hypersensitivity” or “hypersensitivity” and no anaphylactic reactions were reported.

The effect of ADA on the pharmacokinetics of lipegfilgrastim was investigated in a pooled analysis of data from patients with breast cancer and patients with non-small cell lung cancer in the phase III study. Only two patients for whom pharmacokinetic data are available tested positive for ADA, and no decrease in exposure, as indicated by the predicted area under the curve data, to lipegfilgrastim was observed in these patients (data not shown). A pharmacodynamics analysis with a CD34+ endpoint conducted with adult patients for all lipegfilgrastim doses found only two patients with positive ADA response. The CD34+ values for these two patients were similar to those from ADA-negative subjects (data not shown).

## 4. Discussion

The objective of this analysis was to assess the immunogenicity of lipegfilgrastim in patients receiving chemotherapy for breast cancer. In both phase II and phase III studies, the incidence of treatment-emergent ADA was low and was similar between the lipegfilgrastim (phase II: 2/154 = 1.3%; phase III: 1/101 = 1.0%) and pegfilgrastim (phase II: 1/54 = 1.9%; phase III: 1/101 = 1.0%) groups.

Among lipegfilgrastim-treated patients with treatment-emergent ADA, none of the postdose positive samples from either study exhibited NAb activity. Furthermore, there was no apparent impact of treatment-emergent ADA on key measures of clinical efficacy in these patients, including the duration of severe neutropenia or incidence of febrile neutropenia. The limited pharmacokinetic/pharmacodynamic data available for patients positive for ADA suggested no correlation between lipegfilgrastim exposure and ADA positivity.

The results in this study are consistent with literature reporting the immunogenicity of pegfilgrastim, in which a small number (4/521; 0.77%) of pegfilgrastim-treated patients developed treatment-emergent ADA [13]. Similar to the current study, none of these ADA-positive patients showed evidence of NAb activity.

In the current analysis of ADA data from two clinical studies in patients with breast cancer, 10 of 255 (3.9%) lipegfilgrastim-treated patients had positive samples at baseline, a percentage similar to that among pegfilgrastim-treated patients (7/155; 4.5%). Moreover, these patients did not experience an increase in their preexisting antibody response, maintaining relatively low titer levels throughout the course of treatment. Previous studies, again possibly using different assay methodologies, detected anti-pegfilgrastim antibodies in baseline samples from nearly 6% of pegfilgrastim-treated patients with metastatic breast cancer [13]. Therefore, it is not unexpected that some preexisting anti-lipegfilgrastim antibodies were observed at baseline in the current analysis.

This analysis found a low incidence of treatment-emergent ADA in both lipegfilgrastim- and pegfilgrastim-treated patients with breast cancer and treated with doxorubicin and docetaxel. The presence of treatment-emergent ADA did not appear to impact the clinical efficacy of either treatment; this was expected, because none of the detected antibodies in patients with possible treatment-emergent antibodies were neutralizing. Similarly, 11 of 333 patients (3%) developed ADA following filgrastim treatment in clinical studies, and no neutralizing response was observed in the 11 patients [12].

## Figures and Tables

**Table 1:** Summary of patients with ADA-positive samples.

Phase II study									
Patient	Treatment group	Visit							
		BL	C2D1	C3D1	C4D1	D85	D180	D360	ET
*1*	*Lipegfilgrastim*	*Neg*	*Neg*	*Neg*	*Neg*	*Pos*	*NA*	*NA*	*NA*
*2*	*Lipegfilgrastim*	*NA*	*NA*	*NA*	*NA*	*NA*	*NA*	*Pos*	*NA*
3	Lipegfilgrastim	Pos	Pos	Pos	Pos	Pos	Pos	Pos	NA
4	Lipegfilgrastim	Pos	Pos	Pos	Pos	Pos	Pos	Neg	NA
5	Lipegfilgrastim	Pos	Pos	NA	NA	NA	Pos	Pos	Neg
6	Lipegfilgrastim	Pos	NA	NA	NA	NA	NA	NA	NA
7	Lipegfilgrastim	Pos	NA	NA	NA	NA	NA	NA	NA
8	Lipegfilgrastim	Pos	NA	NA	NA	NA	NA	Neg	NA
9	Lipegfilgrastim	Pos	NA	NA	NA	NA	NA	NA	NA
*10*	*Pegfilgrastim*	*NA*	*NA*	*NA*	*NA*	*Pos*	*NA*	*NA*	*NA*
11	Pegfilgrastim	Pos	NA	NA	NA	NA	NA	NA	NA
12	Pegfilgrastim	Pos	NA	NA	NA	NA	NA	NA	NA

Phase III study									
Patient	Treatment group	Visit							
		S	BL	C2D1	C3D1	C4D1	D85	D180	D360

*13*	*Lipegfilgrastim*	*NA*	*NA*	*NA*	*NA*	*NA*	*NA*	*Pos*	*Pos*
14	Lipegfilgrastim	NA	Pos	Pos	Pos	Pos	Neg	Neg	Neg
15	Lipegfilgrastim	NA	Pos	Pos	Neg	Neg	Neg	Neg	Neg
16	Lipegfilgrastim	NA	Pos	NA	NA	NA	NA	NA	NA
*17*	*Pegfilgrastim*	*NA*	*NA*	*NA*	*NA*	*NA*	*NA*	*NA*	*Pos*
18	Pegfilgrastim	NA	Pos	NA	NA	NA	NA	NA	NA
19	Pegfilgrastim	Pos	NA	NA	NA	NA	NA	NA	NA
20	Pegfilgrastim	NA	Pos	NA	NA	NA	NA	NA	NA
21	Pegfilgrastim	NA	Pos	NA	NA	NA	NA	NA	NA
22	Pegfilgrastim	NA	Pos	Neg	NA	NA	NA	NA	NA
23	Pegfilgrastim	NA	Pos	NA	NA	NA	NA	NA	NA

Note: table includes only patients with ADA-positive samples.

ADA: antidrug antibody; BL: baseline; C2D1: cycle 2 day 1; C3D1: cycle 3 day 1; C4D1: cycle 4 day 1; D85: day 85; D180: day 180; D360: day 360; NA: screened negative sample; Neg: confirmed-negative sample; Pos: confirmed-positive sample; S: screening (predose) time point.

Italic font indicates patient with treatment-emergent ADA.

**Table 2:** ADA titer and binding specificity of ADA-positive samples from lipegfilgrastim-treated patients.

Patient	Competitor/titer	Time point						
		BL	C2D1	C3D1	C4D1	D85	D180	D360
Phase II study								

*1*	*Filgrastim*	*NA*	*NA*	*NA*	*NA*	*Neg*	*NA*	*NA*
	*glycoG-CSF*	*NA*	*NA*	*NA*	*NA*	*Neg*	*NA*	*NA*
	*cPEG*	*NA*	*NA*	*NA*	*NA*	*Pos*	*NA*	*NA*
	*Titer*	*NA*	*NA*	*NA*	*NA*	*0.6*	*NA*	*NA*

*2*	*Filgrastim*	*NA*	*NA*	*NA*	*NA*	*NA*	*NA*	*Neg*
	*glycoG-CSF*	*NA*	*NA*	*NA*	*NA*	*NA*	*NA*	*Neg*
	*cPEG*	*NA*	*NA*	*NA*	*NA*	*NA*	*NA*	*Neg*
	*Titer*	*NA*	*NA*	*NA*	*NA*	*NA*	*NA*	*0*

3	Filgrastim	Pos	Pos	Pos	Pos	Pos	Pos	Pos
	glycoG-CSF	Pos	Pos	Pos	Pos	Pos	Pos	Pos
	cPEG	Neg	Neg	Neg	Neg	Neg	Neg	Neg
	Titer	0	0	0	0	0	0	0

4	Filgrastim	Pos	Pos	Pos	Pos	Neg	Pos	NA
	glycoG-CSF	Neg	Neg	Neg	Pos	Pos	Pos	NA
	cPEG	Pos	Pos	Pos	Pos	Pos	Pos	NA
	Titer	0.3	0.9	0.6	0.6	0.6	0.3	NA

5	Filgrastim	NSQ	Neg	NA	NA	NA	Neg	Neg
	glycoG-CSF	NSQ	Neg	NA	NA	NA	Neg	Neg
	cPEG	NSQ	Pos	NA	NA	NA	Pos	Pos
	Titer	NSQ	0	NA	NA	NA	0	0

6	Filgrastim	Neg	NA	NA	NA	NA	NA	NA
	glycoG-CSF	Neg	NA	NA	NA	NA	NA	NA
	cPEG	Neg	NA	NA	NA	NA	NA	NA
	Titer	0.6	NA	NA	NA	NA	NA	NA

7	Filgrastim	Pos	NA	NA	NA	NA	NA	NA
	glycoG-CSF	Neg	NA	NA	NA	NA	NA	NA
	cPEG	Pos	NA	NA	NA	NA	NA	NA
	Titer	1.5	NA	NA	NA	NA	NA	NA

8	Filgrastim	Pos	NA	NA	NA	NA	NA	NA
	glycoG-CSF	Neg	NA	NA	NA	NA	NA	NA
	cPEG	Pos	NA	NA	NA	NA	NA	NA
	Titer	0.3	NA	NA	NA	NA	NA	NA

9	Filgrastim	Pos	NA	NA	NA	NA	NA	NA
	glycoG-CSF	Neg	NA	NA	NA	NA	NA	NA
	cPEG	Pos	NA	NA	NA	NA	NA	NA
	Titer	0	NA	NA	NA	NA	NA	NA

Phase III study								

*13*	*Filgrastim*	*NA*	*NA*	*NA*	*NA*	*NA*	*Pos*	*Pos*
	*glycoG-CSF*	*NA*	*NA*	*NA*	*NA*	*NA*	*Pos*	*Pos*
	*cPEG*	*NA*	*NA*	*NA*	*NA*	*NA*	*Neg*	*Neg*
	*Titer*	*NA*	*NA*	*NA*	*NA*	*NA*	*1.2*	*2.1*

14	Filgrastim	Neg	Neg	Neg	Neg	NA	NA	NA
	glycoG-CSF	Neg	Neg	Neg	Neg	NA	NA	NA
	cPEG	Pos	Pos	Pos	Pos	NA	NA	NA
	Titer	0.6	1.2	0.9	0.6	NA	NA	NA

15	Filgrastim	Pos	Neg	NA	NA	NA	NA	NA
	glycoG-CSF	Pos	Pos	NA	NA	NA	NA	NA
	cPEG	Neg	Neg	NA	NA	NA	NA	NA
	Titer	2.1	1.8	NA	NA	NA	NA	NA

16	Filgrastim	Pos	NA	NA	NA	NA	NA	NA
	glycoG-CSF	Neg	NA	NA	NA	NA	NA	NA
	cPEG	Pos	NA	NA	NA	NA	NA	NA
	Titer	0.9	NA	NA	NA	NA	NA	NA

ADA: antidrug antibody; BL: baseline; C2D1: cycle 2 day 1; C3D1: cycle 3 day 1; C4D1: cycle 4 day 1; cPEG: PEG portion of lipegfilgrastim; D85: day 85; D180: day 180; D360: day 360; glycoG-CSF: glycosylated granulocyte colony-stimulating factor; NA: screened negative sample or confirmed-negative one (not analyzed in characterization assay); Neg: confirmed-negative sample; NSQ: insufficient sample quantity for analysis; Pos: confirmed-positive sample.

Italic font indicates patient with treatment-emergent ADA.

**Table 3:** Evaluation of the potential impact of ADA on efficacy among patients with treatment-emergent ADA.

Treatment	Patient	ADA + time point		DSN (days)	ANC AUC	ANC maximum	ANC nadir
Phase II study							

Pegfilgrastim	10	D85	Cycle 1	0	160.25	9.60	6.8
			Cycle 2	0	212.95	14.90	7.9
			Cycle 3	0	116.25	8.50	3.6
			Cycle 4	0	91.85	7.50	2.4

Lipegfilgrastim	2	D360	Cycle 1	1	154.24	20.39	0.18
			Cycle 2	1	170.20	21.40	0.0
			Cycle 3	0	225.40	27.64	1.25
			Cycle 4	0	202.26	28.42	1.72
	1	D85	Cycle 1	0	127.80	17.00	0.8
			Cycle 2	0	147.25	9.60	2.2
			Cycle 3	0	121.60	8.30	0.7
			Cycle 4	0	179.55	16.70	1.3

Phase III study							

Pegfilgrastim	17	D85	Cycle 1	0	219.88	47.99	0.89
			Cycle 2	0	198.15	13.73	2.2
			Cycle 3	0	302.98	68.32	2.16
			Cycle 4	0	315.63	57.42	4.06

Lipegfilgrastim	13	D360	Cycle 1	2	160.16	26.14	0.25
			Cycle 2	0	257.95	51.92	1.25
			Cycle 3	0	300.24	58.91	1.2
			Cycle 4	0	291.36	68.75	1.45

ADA: antidrug antibody; ANC: absolute neutrophil count; AUC: area under the curve; D85: day 85; D360: day 360; DSN: duration of severe neutropenia.
